# Selections and Abstracts

**Published:** 1914-10

**Authors:** 


### SELECTIONS AND ABSTRACTS

### NOTICE TO MAKERS MEDICINAL PREPARATIONS.

#### Department of Agriculture Discusses Objectionable Labeling for Medicinal Preparations.

In answer to many inquiries as to proper labeling for medicinal preparations to comply with the Food and Drugs Act as amended, the Department of Agriculture, through the Bureau of Chemistry, has issued the following suggestions to makers and proprietors of medicinal preparations:

1. Claims of Therapeutic Effects—A preparation can not be properly designated as a specific cure, remedy or recommended as infallible, sure, certain, reliable or invaluable, or bear other promises of benefit unless the product can as a matter of fact be depended upon to produce the results claimed for it. Before making any such claim the responsible party should carefully consider whether the proposed representations are strictly in harmony with the facts; in other words, whether the medicine in the light of its composition is actually capable of fulfilling the promises made for it. For instance, if the broad representation that the product is a remedy for certain diseases is made, as, for example, by the use of the word "remedy" in the name of the preparation, the article should actually be a remedy for the affections named upon the label under all conditions, irrespective of kind and cause.

2. Indirect Statements—Not only are direct statements and representations of a misleading character objectionable, but any suggestion, hint or insinuation, direct or indirect, or design or device that may tend to convey a misleading impression should be avoided. This applies, for example, to such statements, as "has been widely recommended for," followed by unwarranted therapeutic claims.

3. Indefinite and Sweeping Terms—Representations that are unwarranted on account of indefiniteness of a general sweeping character should be avoided. For example, the statement that a preparation is "for kidney troubles" conveys the impression that the product is useful in the treatment of kidney affections generally. Such a representation is misleading and deceptive unless the medicine in question is actually useful in all of these affections. For this reason it is usually best to avoid terms covering a number of ailments, such as "skin diseases, kidney, liver and bladder affections," etc. Rheumatism, dyspepsia, eczema and the names of many other affections are more or less comprehensive, and their use under some circumstances would be objectionable. For example, a medicine should not be recommended for rheumatism unless it is capable of fulfilling the claims and representations made for it in all kinds of rheumatism. To represent that a medicine is useful for rheumatism, when as a matter of fact it is useful in only one form of rheumatism, would be misleading; such statements as

"for some diseases of the kidney and liver," "for many forms of rheumatism," are objectionable, on account of indefiniteness.

Names like "heart remedy," "kidney pills," "blood purifier," "nerve tonic," "bone liniment," "lung balm," and other terms involving the names of parts of the body are objectionable for similar reasons.

4. TESTIMONIALS—Testimonials, aside from the personal aspect given them by their letter form, hold out a general representation to the public for which the party doing the labeling is held to be responsible. The fact that a testimonial is genuine and honestly represents the opinion of the person writing it does not justify its use if it creates a misleading impression with regard to the results which the medicine will produce.

No statement relative to the therapeutic effects of medicinal products should be made in the form of a "testimonial," which would be regarded as unwarranted if made as a direct statement of the manufacturer.

5. REFUND GUARANTEE—Statements on the labels of drugs guaranteeing them to cure certain diseases or money refunded may be so worded as to be false and fraudulent and to constitute misbranding. Misrepresentations of this kind are not justified by the fact that the purchase price of the article is actually refunded as promised.

---

### ADVANTAGES IN BOTTLING PASTEURIZED MILK WHILE STILL HOT.

### LABORATORY TESTS THAT INDICATE A POSSIBILITY OF USING THIS METHOD TO ADVANTAGE.

Investigators in the United States Department of Agriculture have found that the process of bottling pasteurized milk while still hot has several advantages which make it seem probable that this method would prove both economical and efficacious when practiced on a commercial scale. In an article printed by permission of the Secretary of Agriculture in the

Journal of Infectious Diseases, the authors declare that this method results in bacterial reductions as great as, or even greater than, by pasteurization in bottles.

The principal advantage of the latter method for the ordinary systems in commercial use is the impossibility of the milk becoming contaminated again while being bottled. There is also some saving of milk, because there is no loss from evaporation. On the other hand, when milk is pasteurized in bottles, it is customary to cool the bottles by placing them in cold water. This necessitates the use of absolutely water-tight caps, otherwise some of the cold water is likely to find its way into the milk bottles, and even a very slight leak may result in contamination. Water-proof caps are not only expensive, but care is essential to see that they actually are waterproof, and moreover, bottles with chipped or otherwise damaged tops cannot be used, no matter how nearly perfect the cap may be.

Laboratory experiments conducted by the investigators indicate that milk may be pasteurized, bottled hot, capped with ordinary cardboard caps, and cooled by a blast of cold air economically and with very satisfactory bacterial reductions. The air cooling process requires a somewhat longer time than cooling by water, but in the laboratory it was found that thoroughly pasteurized milk, bottled immediately, could be cooled slowly without increasing the bacterial content. Whether or not the experience of the laboratory will be found true in commercial practice, remains to be seen. The Department of Agriculture, it is announced, will conduct experiments with a view to determining this important point.

Before the milk is poured into them, the bottles should be steamed for two minutes, the authors are careful to point out. This removes all danger of infecting the milk from the bottles, and is another advantage that this new method possesses.

### THE MANAGEMENT OF BREAST FEEDING.

By Eric Pritchard, M. A., M. D., (Oxon); M. R. C. P. (London).

Physician to the Queen's Hospital for Children. Physician to Out Patients, City of London Hospital for Diseases of the Chest (Victoria Park). Honorary Physician for Infant Consultations, St. Marylebone General Dispensary, Etc.

I have already dealt somewhat fully with the details concerned in the "establishment of lactation," I now propose to consider other matters connected with its subsequent maintenance and the general management of breast-feeding up till the time of weaning.

It will be unnecessary for me to allude further to the necessity for long intervals between feeding and for giving an extended period of rest during the night, but I would refer to some very interesting observations made by Dr. Maynard Ladd (8) of Harvard which show that the stomach requires even a longer period than is usually supposed to become completely empty, sometimes as long as 6 hours.

The management of the quantity of milk supplied is a matter of prime importance, not so much during the first few weeks of life, when the chief desideratum is the establishment of the normal functions of digestion, as from the end of the first month onwards when considerations of nutrition should occupy our attention.

There is a very general belief that, as the infant grows older and larger, there is a corresponding increase in the amount of breast-milk consumed, this is by no means an invariable rule, as my own observations (1) on a large number of infants of the poorer class very clearly prove.

There is indeed a serious risk of breast-fed infants being underfed during the latter months unless precautions are taken to prevent this catastrophe.

I imagine I do not overstate the case when I say that of all the factors in the environment which make or mar the development of the infant, food or feedings is the most important. The feeding requires management, both as regards quality and quantity. It is very difficult to influence the quality of breast-milk, this is for the most part beyond our control, but we can manage the quantity or, at least, we can with very little trouble make ourselves acquainted with the exact quantity of food consumed, especially when we are dealing with infants of well-to-do parents. For many years past I have estimated by means of the "test-feed," in many cases repeatedly performed, the amount of milk consumed by all breast-fed infants brought to my clinics, both at the Queen's Hospital and at the St. Marylebone General Dispensary, and by this means I have gained some experience of the quantitative variations in the milk supply of different women. These variations are so bewildering and often so unexpected that I now fully realize that it is merely playing with an important matter to attempt to treat breast-fed infants for nutritional and other disturbances until this primary element in the diagnosis has been settled. I have, indeed, had some very strange experiences which all go to show how utterly futile it is to attempt to decide this question without the confirmatory evidence of the "test-feed." Many times have I though that the infant was being starved, when in reality it was being "over-fed," and often I have thought that "over-feeding" was the trouble when events have proved that the symptoms were due to starvation. There are indeed certain clinical tests by which we may distinguish between under-feeding and over-feeding and I do not hesitate to refer to these very obvious aids to diagnosis, because I notice that they are systematically ignored even by practitioners of wide experience

The cardinal symptoms of under-feeding are

(1) Loss of weight, or at least failure to gain weight at the normal rate.

(2) Constipation, or in case of extreme starvation the passage of small mucous stools, and

(3) A limited excretion of urine (oliguria).

Whereas the cardinal symptoms of over-feeding are

(1) At first unduly large increments in weight succeeded by a period of stationary or falling weight.

(2) The passage of large bulky stools and the presence of redness of the buttocks. Constipation sometimes develops, but the motions are large when passed.

(3) The passage of a large quantity of water (polyuria).

(4) Sweating and vascular dilation of the capillaries of the face, and

(5) Rapid breathing.

In addition to these cardinal symptoms there are other means by which we may distinguish between starvation and superalimentation; for instance the condition of the mother's breasts conveys very important information.

Breasts which are full and large before feeding, and small and flaccid after feeding presumably afford a good supply, whereas small breasts which do not perceptibly diminish in size after feeding as a rule cannot be credited with a liberal secretion, but no matter how carefully we weigh or attempt to piece together such fragments of diagnostic evidence, it is impossible to rely on any other evidence than that of the "test-feed."

To show how very misleading the subjective sensations of the mother may be, and how utterly worthless her own opinions as to the amount of milk she may supply, I herewith give the particulars of two interesting and illustrative cases.

Case 1.—T. W. (Record No. 2913). A male infant 2 months old was brought to the Queen's Hospital, Feb. 10th, 1913, for continuous screaming. The weight at birth was not noted, but the baby was reported to have been of average size; the weight on being brought to the hospital was 10 lbs. So presumably the infant had increased in weight some 2 or 3 lbs. in the two months, and therefore could not have been systematically starved. For some days past the infant had been constipated, and had passed a very small quantity of water. The mother's breasts were of normal size and appeared well developed, but milk could only be expressed with difficulty. As the infant had been recently put to the breats I deferred a "test-feed" till the next visit, giving the mother instructions not to feed the infant for 3 hours before attending at the hospital. In the meantime feeling fairly confident from the symptoms that the infant was being starved, whatever might have taken place at an earlier date, I told the mother to give the infant 1 teaspoonful of condensed milk and two tablespoonfuls of water after each breast-feeding. Owing to a mistake on the part of the mother, it was not possible to give a test-feed when the infant was brought to the hospital a week later, but in the interval the infant had gained 2 lbs. 4½ ozs. in weight, the largest increment I have seen registered in one week; in fact, no infant could possibly show such an increment unless it had been very seriously starved for some time previously. I have doubt that the infant's tissues were very dry and that this extraordinary increase in weight was chiefly due to their rehydration. Two days later a proper "test-feed" was given, but this showed that the infant weighed the same after the feeding as it did before it was put to the breast, a negative result which was confirmed in the following week by means of a second test-feed.

It is interesting to note that on the occasion of each of these "test-feeds" the mother told me, in answer to my enquiry, that the infant had had a good feed, and that she felt sure it obtained quite as much as it had during the early days of life when it was making good progress, and did not scream.

Seven days later another "test-feed" was given but the result still proved that the breasts were dry. In the interim, however, the infant had gained further weight and was now 13 lbs. 4 ozs. The mother was apparently so satisfied with the result of the artificial feeding that she ceased her attendance at the hospital and I have not seen the infant since.

This result illustrates one of the great dangers of supplementary feeding with condensed milk; owing to the sweetness of the milk the infants take so kindly to the food that they do not seem to apply much energy to the task of obtaining milk from a relatively dry breast. Moreover, the mothers are so pleased with the immediate results, that they are only too glad to relinquish breast-feeding with all its attendant troubles and disappointments. I have very little doubt that the infant above referred to grew into a fat, contented, flabby and unhealthy baby, like the rest of its kind who are fed on this most unsatisfactory food.

I have great faith in condensed milk as an easy stepping-stone to better things, but as a permanent bridge between the period of early weaning and the solid food stage, it is full of temptation and dangers. The three main faults in condensed milk, at any rate the ordinary sweetened variety, are (1) that it contains a great excess of sugar, (2) that it is too easily digested and does not develop the functions of digestion and (3) that it is a dead food which contains no antiscorbutic elements (vitamines).

The following cases illustrate the value of the "test-feed" from a totally different point of view.

Case 2.—W. W. (Record No. 3340). A male infant 6 months old and weighing 16 lbs. 6 ozs. was brought to the Queen's Hospital for constipation and glands in the neck. The infant was fed every two hours and the mother was under the impression that she had not sufficient milk. I gathered from the mother's account of the condition of the napkins that the infant passed a large quantity of water, and from the appearance of the breasts and the ease with which milk could be expressed, I felt convinced that from whatever else the infant might be suffering, its present condition was not due to want of milk. I gave the mother the usual instructions and two days later a "test-feed" was given at the hospital; this proved that the amount of milk which the infant extracted from the breast was very nearly seven ounces, a quantity which in my experience of hospital practice in the East End of London is a record amount. This infant was clearly suffering from over-feeding, and on reflection the symptoms appeared to be by no means inconsistent with this explanation. Constipation is not an uncommon accompaniment of superalimentation, but in this particular case, as I subsequently discovered, the constipation was due to the abuse of soap-and-water enemata. As I so frequently point out at my clinics, there are few more aggravating causes of constipation in infant than the early dislocation of the normal rectal reflex by the administration of enemata, glycerine suppositories and powerful aperients. I treated this case by extending the intervals between feeding, by reducing the amount of clothing and by promoting the physiological demand for food in the many ways I have already mentioned. Thus I overcame the evil consequences of over-feeding and the infant soon improved in condition. It at once put on weight and was in perfectly good health when the mother ceased attending at the hospital.

I could easily multiply instances both of over-feeding and of under-feeding which have been revealed and cured owing to the instrumentality of the "test-feed," but no degree of repetition would be so convincing as a short experience of the method in an infant clinic. Personally I cannot understand how any physician can expect to treat breast-fed infants successfully unless he first takes the precaution of acquainting himself with the amount of food the infant consumes.

I imagine that the two really important factors in the infant's environment are the quantity and the quality of the food. It is very difficult to obtain accurate information with respect to the quality of breast-milk, for chemical analysis shows but little, but it is quite easy to obtain precise information with respect to the quantity, and I consider that any physician who fails to take this precaution does not do his duty by his patient.

Although for the last 10 years I have practically never omitted to gauge as accurately as I have been able, the amount of milk which has been consumed by every breast-fed infant concerning whom I have been consulted, I freely admit that even with this experience I am unable to form a reliable estimation by any clinical test with which I am acquainted except the "test-feed."

I have elsewhere explained that, within certain wide limits, the supply of breast milk is correlated to the demands of the infant, that is to say, other things being equal, the more hungry and lusty the infant, the larger will be the amount of milk afforded by the breasts, in other words, there is some parallelism between the intensity of the stimulation and the reflex response. But this physiological correlation does not obtain under all conditions, its fulfilment demands the existence of normal conditions in the secretory apparatus and its nervous mechanisms, a very large assumption. For instance the casual presence of a crack in the nipple may induce painful impressions, instead of normal stimulation, and such painful impressions may suppress rather than excite secretion in the mammary glands, or psychological disturbances in the mother may interfere with the normal liberation of the reflex response. Again, the infant itself is, like the mother, subject to psychological disturbances, and for some quite unforeseen reason may refuse to suck and apply the required stimulus. Suggestion plays a most important part in the actions of an infant. For instance, one or two disappointments at the breast may shake its faith, and it may refuse to make what it considers to be wasted efforts. Or again, sometimes the effort of sucking may initiate painful sensations in the abdomen, due to spasms or contraction of sphincters or muscular bands; énterospasms and other varieties of incoordinated peristalsis often cause excruciating pain, and when once the infant associates such experiences with the act of taking the breast, it may resolutely refuse to be a party to any repetition of the attempt.

The truth is that breast-feeding requires a great deal of management if it is to be a complete success. It is a mistake to underrate its difficulties or to exaggerate its merits.

One of the most important matters in conection with breast-feeding is to know when and how to supplement an inadequate supply with artificial feedings. In this connection two factors must be considered. Firstly, how much food ought any particular infant to receive in the 24 hours. And secondly, how much does it actually derive from the mother's breasts. As regards the first factor I must refer to what I have already said in my lecture on the quantitative requirements of infants. If it is found by means of the test-feed that the quantity of milk supplied by the mother falls materially short of this amount, then the deficit must be made good by supplementary feedings. It is very difficult to increase the natural supply of any expedient known to science although plenty of gratuitious advice is always forthcoming from amateur sources.

Galactogogues which directly stimulate the flow are unknown, and food makes very little impression. Starving women afford quite a good supply as we know from the experiences of the Siege of Paris in 1870, and the great cotton famine in Lancashire some years prior to this date. On both of these occasions the infant mortality was peculiarly low, the infants benefitted rather than the reverse from starvation of the mothers. I had an interesting illustration of the insignificant influence of starvation on the mother's milk a few days ago, when a woman, whom I had know for some years, came to my infant consultation with a 3 months' old baby whom she was suckling. This woman was seriously suffering from the effects of inanition, and yet the baby was plump and well, increasing normally in weight, and appearing quite well in other respects.

The test-feed showed that the amount of milk obtained at a feeding was ample. Dr. Leslie Duncan (2) has made some interesting observations on the influence of dinners supplied by necessitous nursing mothers in Birmingham. The extra food supplied in this way to half-starved women had a distinct influence on the fat content of the milk, but in no other way appeared to influence its quality, although the nutrition of the mothers materially improved in consequence of the extra food.

This has been my own experience also, although I have not had an opportunity of confirming my clinical experience by many actual analyses of the milk.

On the other hand, if a nursing mother is already obtaining a sufficiency of food, no good purpose will be subserved by amplifying her already adequate dietary. Nursing stout, gruel and cod liver oil often do more harm than good by making the mother bilious and thus upsetting the general equilibrium on which a good mammary secretion so largely depends: I would, however, say one word in favor of iron as an adjuvant to the nutrition of the nursing mother; it is certainly a drug which may improve the quality of the milk. At the best of times breast-milk is not too rich in iron, and it may show a noticeable deficiency if the mother has been depleted of iron by hemorrhages or other conditions which dispose to anemia. Infants very soon show the effects of iron starvation, and their general condition of nutrition may under certain circumstances greatly improve when iron is administered to the mothers. I make a common practice of supplying nursing mothers with 15 to 60 grains of carbonate of iron in the 24 hours, and I generally notice that the quality of the milk—as estimated by the nutrition of the infants, improves. Seeing that by ordinary means we cannot increase the quantity of the natural supply, we must compensate for a shortage of breast-milk by giving supplementary feedings; and it is certainly my experience that some of the best results which I have seen have been in the case of infants fed by the combined method. The supplementary feedings can be given in various ways, either at the beginning or at the end of a breast feeding, or as an occasional substitute for it, that is to say by alternating breast and bottle-feedings, or by substituting 1, 2, 3 or more bottles for the breast.

I would not like to lay down any hard and fast rules. Each case must be judged on its own merits. Sometimes on enquiry, or in consequence of a test-feed, we find that at one particular hour in the day the mother's milk materially fails. If this is so, this hour should be chosen for the supplementary bottle—I find from experience that the late afternoon feeds are the poorest in quantity, just as the first morning feed, after the long rest at night is usually the largest.

Sometimes I find it an excellent plan to give a small supplementary feed at the end of each feeding. I have had some very extraordinary cases both at the Queen's Hospital and at the St. Marylebone General Dispensary, in which the addition of an insignificant quantity of extra food has produced quite a disproportionate change in the infant. Infants whose weight may have been stationary for weeks or months may increase a pound or more in weight in quite a short time by the addition of a few ounces of cow's milk in the 24 hours.

It is naturally of some importance to supply the right food in the right amount when recourse is had to supplementary feedings. The amount of food to be given by hand will depend on the quantity of breast-milk already taken by the infant. It is unwise to augment the quantity too suddenly or too drastically. The danger of overloading an unprepared stomach is very great.

If an infant obtains say 15 ozs. of breast-milk from the mother in the 24 hours, when according to the theoretical estimation the amount should be 10 ounces more, we must consider in what way to supply the deficiency. If we find after giving a few test-feeds that sometimes the infant obtains a large quantity and sometimes a small, it may be quite safe to give the supplementary food in comparatively large quantities, say in doses of 3, 4 or 5 ounces. On the other hand, if the infant appears to obtain its total supply in small and constantly regular quantities, it may be better to distribute the supplemetnary food in the form of small and increasing addendums to each feed, but as I say, there is no rule, and each problem must be solved by individual consideration of the attending difficulties.

I find among the poor the simplest and most practical method of supplementing a defective breast supply, is to order a quarter, a half or a whole teaspoonful of condensed milk to be given after each breast feeding, the food to be given in a spoon without any previous dilution; this obviates the possible danger of overdistending a stomach already full of a thin and poor milk, for it must be remembered that, infants are often underfed, although the quantity of breast-milk may be ample. This is especially the case toward the end of lactation, when the mother has become exhausted from prolonged suckling and the milk becomes thin.

I feel in this lecture that I have omitted a great deal that ought to be pointed out in any complete account of breast-feeding, my only defence for these omissions is that I have thought it advisable to husband time and space and confine myself as far as possible to the sides of the question which are generally omitted in text-books and other works dealing with the subject.

BIBLIOGRAPHY.

(1) Breast Feeding and the Value of the Test-Feed. The Lancet, Sept. 2, 1911.

(2) Report on Infant Mortality in St. George's and St. Stephen's Wards. Birmingham, 1913. Printers, Hudson & Son.

(3) Breast-feeding of Infants. Medical Press and Circular, July 2, 1913.

(4) Breast-Feeding; Its Management and Mismanagement. The Lancet, June 14, 1913, page 1657.

(5) The Nursling. Pierre Budin. English translation, 1st Edition, page 37.

(6) H. C. Cameron. The Lancet, Sept. 27, 1913, page 911.

(7) Professor Hans Reitchel, Lehrbuchf. Kinderheilk, April 1912, page 403.

(8) The Influence of Variations of Diet upon Gastric Mobility in Infants. Archives of Pediatrics, 1913, p. 740.

—American Medicine.

---

### THE TREATMENT OF SYPHILIS OF THE NERVOUS SYSTEM.

JOHN A. FORDYCE, M. D., PROFESSOR OF DERMATOLOGY AND SYPHILOLOGY, COLUMBIA UNIVERSITY, COLLEGE OF PHYSICIANS AND SURGEONS.

While it was recognized soon after syphilis became pandemic that the nervous system played a part in the morbid process, it appears that Knorre was the first, in 1849, to call attention to the fact that paralyses might occur in constitutional syphilis soon after or synchronously with the earliest symptoms. He cited five cases in three of which paralysis occurred three months after infection. Since then numerous publications by neurologists and syphilologists have appeared giving clinical evidence of the early involvement of the nervous system. Nonne has observed cerebral syphilis developing four months after infection and mentions a case occurring simultaneously with the roseola. Leudt reported brain lues in two old persons three months after the appearance of the initial lesion. Mott has observed meningitis even before the primary sore was healed, and he is sponsor for the statement that the most severe cases of brain and spinal syphilis occur within the first twelve months. Naunyn collected 209 cases from the literature, to which he added forty-five of his own. From his analysis he came to the conclusion that infection of the nervous system took place in the majority of instances during the first year, after which there was a decrease in frequency. Brau's statistics, made up of 100 cases in which a history was obtainable, showed that in nearly one-half the nervous system was involved within the first year. In Fournier's clinic twenty-six cases were shown to have developed within from three to six months after infection. Hutchinson's fifteen cases of spinal syphilis appeared from six months to three years after infection, while Savard found that out of seventy-seven cases of myelitis, twenty-six appeared from six to eight months after the chancre. These observations on the clinical side have been considerably augmented during the past few years by laboratory findings.

In 1903 Ravaut undertook a systematic examination of the spinal fluid in secondary syphilis and found abnormal changes in about 70 per cent of the cases. At the same time he made note of an apparent parallelism between the type of cutaneous eruption and the findings in the spinal fluid, having been impressed by the more frequent occurrence of abnormalities with papular, pigmentary and psoriasiform lesions than with the roseola. Plaut also obtained a rather high percentage; namely, a definite pleocytosis in one-third of the cases and border-line results in another third, leaving only one-third negative. Nonne and Mantoux placed their positive findings at 40 per cent. From the examinations of Altmann and Dreyfus, chemocytologic changes were present in about 80 per cent of patients in the secondary stage with remains of the initial lesion. Gennerich found positive findings in about 90 per cent. He is of the opinion that all early constitutional cases, that is, those in which the Wassermann has become positive, will after an injection of salvarsan or mercury develop an increase in lymphocytes and globulin. In other words, a provocative or Herxheimer reaction will be obtained in the fluid. This concurs with the studies of Ravaut, Levy-Bing, Duroex and Dogny. Altmann and Dreyfus made similar observations. One of their cases in the initial stage with a negative blood and spinal fluid shoowed sixteen days after treatment with 1gm. salvarsan, fifteen cells. Another with a negative blood and fluid sixteen days after 1.4 gm. salvarsan had a marked increase in pressure. Two months later, during which time an additional 1.5 gm. had been given, twelve cells were found. Another patient in the early secondary stage with normal fluid was given 0.4 gm. salvarsan. Seven weeks later the patient had facial and acoustic paralysis with 180 cells and a strongly positive Wassermann. This was a characteristic neurorecidive, a number of which cases were reported early in the salvarsan era, but are no longer met under our more intensive methods of treatment.

My own investigations up to the present time do not coincide with the high percentage of anomalies reported by other observers. In the absence of clinical symptoms pointing to involvement of the central nervous system in the early secondary stage, I have found abnormalities in the spinal fluid in less than 20 per cent. The headaches of the early secondary period are frequently unattended by changes, and appear to be due to a hypertension rather than a meningitis. In the later secondary and tertiary periods the cephalalgia is accompanied more usually by a lymphocytosis and other findings and is the precursor of cerebral accidents. I still have this subject under investigation and will report on it later. My view at this time, however, is that too much importance has been attached to trivial changes, as differences in pressure or slight increase in globulin, and that probably no larger percentage show marked anomalies than later develop definite nerve changes. That such cases may be the subject for years of a chronic meningitis without objective signs has been shown by the researches of Vincent, who was able to follow for a number of years patients with marked fluid changes and no clinical symptoms, who later developed severe organic changes in the central nervous system, some paresis. It therefore seems probable that cases of syphilis of the nervous system are recruited from those with a chronic meningitis or latent residua, for we know that the older the infection becomes the greater is the tendency to localization or tertiarism. Whether or not a new infection or dissemination of spirochetes can take place from an old focus we must submit for future enlightenment. In this connection may be mentioned the positive inoculation obtained by Uhlenhuth and Mulzer, in a rabbit with the blood of a woman with latent syphilis, who had been delivered shortly before of a syphilitic child. Her infection dated back four years.

Clinical observations have taught us that only a minority of syphilitic individuals develop obtrusive nervous symptoms. Even under the old and inadequate methods of treatment the percentage is not very high. This may be accounted for partly by less complete methods of diagnosis, as the majority of statistics were compiled before the application of the Wassermann reaction or the lumbar puncture. Hjelmann placed his figures of cerebral syphilis at from fifteen to twenty-five per thousand. Raumont found that of 3,400 cases, 290 suffered from syphilis of the nervous system. Fournier's investigations of 4,329 cases of tertiary syphilis elicited diverse nervous manifestations in 1,085, of which 416 showed involvement of the cerebrospinal axis with more or less diffuse lesions. Pick and Bandler followed the fate of 2,066 cases in the Prague clinic between the years 1879 and 1899 and found that 4.25 per cent had developed parasyphilis. Most valuable are the statistics of Mattauchek and Pilcz. In an admirable and painstaking manner they investigated the course of the disease in 4,134 officers in the Austrian army who had been infected during the years 1880 to 1900, and found that 198 developed paresis and 113 tabes, not counting those who later became paretic. Of the total number of tabetics 11.93 per cent developed paresis; 8.18 per cent had optic atrophy, not including the cases of taboparesis; 132 patients suffered from cerebrospinal lues, most frequently the endarteritic form, and eighty other cases showed various psychoses.

Dreyfus examined 104 patients with latent syphilis, none of whom had symptoms pointing to involvement of the central nervous system. He found 77 per cent normal. Of the remaining 23 per cent, only 12 per cent showed marked changes. Inferring that the majority of patients suffer from meningeal involvement at the beginning of the infection, we must also infer that in a large percentage, cure takes place in spite of lack of or insufficient treatment, for in the cases under discussion Dreyfus mentions that treatment had been only symptomatic and far from adequate according to our present notions.

This leads us to another angle of the problem, namely, the question of "lues nervosa." It has long been the belief of syphilologists and neurologists that in nerve syphilis we are dealing with a different form of the infection. This had its origin in the observation of conjugal and family groups of metasyphilis as well as in persons infected at a common source. The question has been whether the factors which determine the later development of nervous syphilis reside in the infecting agent or in the host. Nichols has recently expressed the opinion that such spirochetes possess a highly invasive power rather than a neurotropic action. His deduction rests on the results he obtained with a strain isolated from the nervous system. He found that they had a thicker form and a shorter incubations period, producing in rabbits hard lesions with necrotic centers and showing a tendency to generalize with lesions of the skin and eye, following local inoculation of the scrotum and testicle. Steiner's experiments with rabbits showed that in only a few cases the nervous systems are involved, in some the lesions are in the brain, in others, in the cord. Dorflein's work with the spirochetes or relapsing fever is perhaps also pertinent in showing that species, geographically separated, possess different biologic properties which are not distinguishable morphologically.

I have many times noted the involvement of the nervous system coincidently in husband and wife, and have at present under observation a woman with cerebrospinal syphilis whose husband had a paraplegia, a man with paresis whose child had hydrocephalus and a couple with taboparesis. Fischer found among 500 paretics conjugal metasyphilis in 10.2 per cent. In Fischer's analysis, husband and wife had general paralysis 26 and tabes 31 times; the husband tabes and the wife paresis 4 times, vice versa 15 times; both cerebrospinal syphilis 4 times; husband tabes and wife cerebrospinal syphilis one, and husband paresis and wife cerebrospinal syphilis once. Of the hereditary cases he found that when one parent had paresis the child had paresis 12 times and tabes 5 times. When one parent had tabes the child had tabes 10 times and paresis 3 times. It has been noted that when the types of conjugal metasyphilis are dissimilar, the man more often suffers from paresis and the woman from tabes.

Experience has demonstrated that many luetic affections of the nervous system are amenable to the treatment ordinarily employed, that is, mercury by injection or inunctions or by a combination of mercury with potassium iodide internally. The lesions which are responsive to treatment of this kind are certain types of endarteritis, localized gummas, periostitis involving the meninges and disseminated forms of meningitis. There are, however, some forms of gummas and chronic meningitis with severe headaches and endarteritis with symptoms, such as irritability, loss of memory, and inability to concentrate, which do not yield to the old treatment, as well as the types of syphilis formerly described under the names of parasyphilis or metasyphilis. The latter were so resistant to treatment that it had long led to the belief that they were only indirectly related to the infection—rather the result than the direct effect of the action of the organisms. The investigations of Noguchi and Moore demonstrating the presence of spirochetes in the lesions of paresis and tabes have, however, definitely placed these so-called parasyphilitic affections among the active lesions of syphilis.

In the treatment of syphilis of the nervous system the important points to emphasize are first, accurate diagnosis with serologic findings, second, necessity of prolonged treatment, third, the avoidance of too large initial doses until the susceptibility of the patient has been determined, and fourth, persistence in treatment in spite of absence of amelioration following the first few injections.

The criticism of the results of treatment of the nervous system by writers who have noted no improvement or an aggravation of symptoms depends on the superficial way in which it has been carried out. No judgment can be rendered until patients have been subjected to a sufficiently long treatment to influence permanently the pathologic changes in the cerebrospinal fluid. They must be kept under continuous observation for a period of years, and until the four phases of Nonne have become negative, no assurance can be given against relapses. In other words, since the infection of the nervous system is more difficult to reach, and hence more persistent, the treatment should be carefully controlled not only by clinical results but also by lumbar puncture. We cannot form any conception of the activity of the process from the symptoms alone. The latter sometimes are out of all proportion to the laboratory findings. There are at present under my care two patients in both of whom the only complaint was headache and a feeling of malaise. In the one a cell-count of 314 with double plus globulin and positive Wassermann to 0.2 cc. was found. In the other there was present a pleocytosis of 746 cells, a great excess of globulin and a positive reaction to 0.025 c. c.

It is not possible at present to give a characteristic serologic picture for cerebrospinal lues, tabes and paresis. Bearing in mind that there are transitional or border-land forms, it is necessary to supplement laboratory findings with careful and minute clinical examinations. To obtain a proper conception of the serologic findings in early tabes, repeated examination of the fluid should be made in all patients presenting symptoms pointing to involvement of the nervous system. Many cases of tabes give a previous history of oculomotor paralysis, facial palsy, headaches, somnolency, irregularity of the pupils or other evidence of meningitis of the base. In other cases in which the process is limited to the lower cord, these early symptoms are lacking. With the acquisition of further data we may be able from a group of symptoms and spinal fluid findings to foretell the subsequent development of tabes. Gummas or endarteritic changes in the nervous system frequently occur, but recovery takes place without secondary tabes or paresis, while cases with meningitis of the base may be followed by either of these conditions. The closer these conditions are studied from a serologic and anatomic point of view, the less likelihood is there that they are due to a primary neuron degeneration. Nonne is of the opinion that they are the result of a meningitis and refers to the work of Schroder, Meyer and Oppenheim demonstrating inflammatory changes in the pia and connective tissue passing from it to the blood-vessels. Nageotte also found a true inflammation with lymphocytes and plasma-cell infiltration about the root-fibers, as well as inflammatory changes in the arteries and veins. Stargardt likewise demonstrated a true inflammation of the sheath and endoneurium of the optic nerve in cases of tabes and paresis. It is possible for the specific infection to be limited to the cerebrospinal fluid, the blood being entirely free from the infection. This is proved by the negative findings in the blood and the positive ones in the fluid. It has also been demonstrated that the interchange between the blood and the spinal fluid is so slight that drugs introduced by the blood-stream diffuse themselves very little if at all in the spinal fluid. This fact explains the resistance of certain types of syphilis to the ordinary methods of treatment. Why this is so in one case and not in another is difficult to explain, unless we assume a more pronounced endarteritis in one case than in another or greater permeability in the choroid plexus. No matter what the theoretical considerations are, practical results show that the cells and other abnormalities can be influenced by intravenous treatment, although this takes place more slowly than with the direct method. That the latter is not only local in its action is shown by the change of a positive to a negative reaction in the serum by the exhibition of this treatment only.

The intraspinal method has placed the treatment of syphilis on a more accurate and scientific basis. As first applied by Swift and Ellis, it consisted of injecting into the subarachnoid space salvarsanized serum, that is, the serum from a patient treated intravenously with the drug one hour before. Diluted with normal saline it is given in strengths varying from 40 to 100 per cent. This method has been modified by Dr. Hanson S. Ogilvie by incorporating salvarsan with human serum direct in vitro, and has the advantage of standardizing the dose. For the procedure his technic is as follows:

Fifty c. c. of blood are drawn into a centrifuge bottle and centrifuged twice. It is important to have the serum clear and free from fibrin and blood-cells. To obtain the requisite amount of the drug, old salvarsan is mixed in the usual way in the proportion of 0.1 gm. to 40 c. c. of fluid, care being taken not to over alkalinize; 0.4 c. c. of this solution is the equivalent of 1 mg., and is taken as the standard for measuring the dosage. For this purpose a 1 c. c. pipet graduated in hundredths should be employed. The desired amount of salvarsan is added to from 12 to 15 c. c. of the serum, shaken to and fro to mix thoroughly, and then placed in the incubator at 37 C. (98.6 F.) for one hour, after which it is inactivated for half an hour at 56 C. (132.8 F.). The latter is a most important step in the technic, as Swift and Ellis demonstrated that the spirocheticidal properties of the serum were markedly increased by heating.

In experimenting with trypanosomes, Stuhmer found that the curative action of unheated salvarsanized serum lasted twenty-four hours, and its protective action forty-eight hours, while in the heated serum the curative action was still present at the end of three days and the protective action four days. In comparing the relative values of salvarsan and neosalvarsan, he found that whereas the former was still active after forty-eight hours and often seventy-two hours, neosalvarsan lost its parasiticidal powers after twenty-four hours.

Salvarsanized serum prepared according to the method of Ogilvie must be used fresh, that is, within three hours of the time that it is made up. Patients should be prepared for its administration as for the intravenous injection, with a laxative the night before and only a light meal two hours prior to the treatment.

A lumbar puncture is made and an amount of fluid equivalent to the amount introduced is withdrawn. While the needle is in situ, the barrel of a Luer syringe is connected by means of a piece of rubber tubing. Spinal fluid is allowed to fill this to expel the air and the serum is then permitted to flow in by gravity. After its administration the patient should lie flat without any pillows, the foot of the bed being elevated for several hours. He must be kept in bed for at least twenty-four hours, in some cases forty-eight or seventy-two hours. Failure to do this may result in unpleasant symptoms, as pain in the extremities, headache, anesthesia, and bladder and rectal paresis.

The proper dose is still in a stage of evolution and a note of warning must be sounded against employing too large amounts. From my experience during the last months I feel that the limits of safety lie within 0.5 mg. It is better to begin with a dose 0.25 mg., repeating or gradually increasing according to the tolerance of the patient. The intervals should be two weeks or longer. In a number of patients, when larger doses were given and the intervals were only a week apart, unpleasant by-effects were noted. These may come on within a few hours or be delayed for six days and may persist several weeks. The symptoms consist of root-pains, numbness, bladder and rectal paresis in some, irritability of the bladder in others, and ataxia. The numbness in some cases involved the whole lower portion of the body, in others the gluteal region and the legs with areas of hyperesthesia on the inner thighs.

On the Continent, where neosalvarsan is employed intraspinally, Marinesco and Minea and Ravaut have given as much as 12 mg. at a dose. These large doses were followed in a number of cases by severe reactions.

On account of the technical difficulties connected with sub-arachnoid injection it is well to inaugurate the treatment of nerve syphilis with a few mercurial injections and follow this up by a course of intravenous injections of salvarsan, beginning with a dose of not more than 0.25 or 0.3 gm. and repeating these injections at varying intervals of from one to two weeks. At the end of six or eight such injections lumbar puncture should be repeated; and if a marked effect has not been obtained either clinically or serologically, a course of intraspinal injections should be seriously considered, the number and intervals to be governed by the reaction produced. Should at any time symptoms of irritation manifest themselves, the patient should rest until they entirely disappear.

In the types of disease in which the symptoms are urgent and an immediate effect is desired, there can be no objection to beginning the intraspinal treatment without the antecedent intravenous employment of the drug, but better results are obtained by combining the two routes of medication. It must be borne in mind that a definite plan of treatment has not yet been formulated but that we are in a state of developing a method which seems to offer far more hope of influencing these resistant forms of syphilis of the nervous system than any heretofore employed.

Cases of tabes which are rather acute in their development, and have a high pleocytosis and positive Wassermann are advantageously treated by the combined use of mercury with the method outlined. In those accompanied by marked degenerative changes mercury may hasten the process. It therefore must be employed only after a careful analysis of the symptoms and serologic findings. An indication for intraspinal treatment alone would seem to be present in those cases in which we have a negative Wassermann in the blood and a positive one in the cerebrospinal fluid.

As I believe the results obtained with neosalvarsan are inferior to those obtained with salvarsan, I have practically given up the use of the former. It has been shown by Stuhmer that, owing to its quicker elimination, its action is much shorter than that of salvarsan. While in each the maximum effect is produced within the first twenty-four hours, it practically ceases on the second day in the case of neosalvarsan, whereas in the case of the old he still found active traces at the end of a week.

In estimating the results obtained by the intensive treatment of syphilis of the nervous system one must not forget the influence of the psycologic effect of a new method of treatment and the fact that in many cases of tabes and paresis there are periods of remission covering months or years. Observations of this kind, however, were made before we employed the more precise methods of diagnosis and controlled our treatment by means of the examination of the spinal fluid. Furthermore, many of our most striking results have been obtained in patients who had long-continued and marked symptoms such as girdle sensation, pains and impairment of locomotion. The change in the clinical symptoms as well as the serologic findings would certainly give us strong presumptive evidence that the results were due to an actual improvement in the condition and not a normal remission.

Each case is a problem in itself and must be approached in an individual manner. Different pathologic conditions are present, so that while on the one hand we have an active process, on the other there is a degenerative one, complicated perhaps with an endarteritis, softening or scar formation. Symptoms which are due to interference with nutrition or pressure we cannot expect to influence.

When salvarsan is employed it should be done so intensively and combined with mercury. The criticism of certain writers against the employment of salvarsan in the secondary stage of syphilis is entirely fallacious, for we know that when it is given in the proper manner combined with mercury, the so-called neurorecidives do not occur. In an experience of over two years, during which this method of treatment has been followed, not a single case of nerve recurrence has been observed.

The cases submitted show that it is possible in some instances by intravenous medication alone to influence the clinical and serologic findings, and when this does not take place the only alternative may be intraspinal medication. Until we are more familiar with the effects of serum fortified in vitro, the Swift and Ellis method of salvarsanizing serum in vivo should be the one of choice. In certain cases the serum from a patient so treated may be reinforced with from 0.25 to 0.5 mg. of salvarsan. It must always be borne in mind that the spinal cord reacts intensively to irritants even of a mild grade. When a degenerative process is present the resistance of the cord is much below that of a normal one and the brunt of the irritation falls on the degenerated areas.

I am convinced that the intelligent and energetic treatment of the infection in the early stages properly controlled by the serum reaction would do much to prevent the subsequent development of nerve syphilis. It must, however, be thorough to eliminate the possibility of residual foci, which experience has taught have a predilection for the meninges. The early history of salvarsan shows that these accidents oclur more precipitately after its use than under the older and milder methods of treatment.—Journal A. M. A.

8 West Seventy-Seventh Street.

